# High prevalence of non-accidental trauma among deceased children presenting at Level I trauma centers in the Netherlands

**DOI:** 10.1007/s12024-021-00416-7

**Published:** 2021-11-13

**Authors:** Marie-Louise H. J. Loos, Roel Bakx, Wilma L. J. M. Duijst, Francee Aarts, Ivo de Blaauw, Frank W. Bloemers, Jan A. Ten Bosch, Martina Evers, Alexander P. A. Greeven, Marie-Josée Hondius, Roland L. J. H. van Hooren, Erik Huisman, Jan B. F. Hulscher, Claudia M. G. Keyzer-Dekker, Egbert Krug, Jack Menke, Tatjana Naujocks, Udo J. L. Reijnders, Victor A. de Ridder, W. Richard Spanjersberg, Arianne H. Teeuw, Hilco P. Theeuwes, Will Vervoort-Steenbakkers, Selena de Vries, Ralph de Wit, Rick R. van Rijn, on behalf of the AsAnTe study group, Anne de Boer, Tina Dorn, Esther Edelenbos, J. Carel Goslings, Steven Kooiker, Irma Michielsen, Lia P. G. W. van Sommeren, Annelies Toor, Marjo Affourtit, Jan C. van Ditshuizen, Rene M. H. Wijnen, Dagmar R. J. Kempink, Gert J. H. J. M. Bessems, Tjebbe Hagenaars, Dennis den Hartog, M. A. C. Jansen, J. H. Allema, Floris E. P. Kanters, Annemieke Aalbers-Hiemstra, Saskia Beunder, Arnaud Mulder, Frans Smiers, Rina C. Hartendorf, Audrey A. A. Fiddelers, Birgit Levelink, Martijn Poeze, Gisela de Heus, Vidija Soerdjbalie-Maikoe, Michael J. R. Edwards, Tjarda N. Tromp, Benn Beuker, Inge H. F. Reininga, Klaus Wendt, Stasja J. G. Aspers, Elise M. van de Putte

**Affiliations:** 1 Amsterdam UMC, Department of Paediatric Surgery, Emma Children’s Hospital, Paediatric Surgical Centre Amsterdam, University of Amsterdam & Vrije Universiteit Amsterdam, Amsterdam, the Netherlands; 2 Department of Forensic Medicine, GGD IJsselland, Zwolle, the Netherlands; 3 Criminal Law and Criminology, Faculty of Law, Maastricht University, Maastricht, the Netherlands; 4 Department of Forensic Medicine, GGD Nijmegen, Nijmegen, the Netherlands; 5 Department of Paediatric Surgery, Radboud University Medical Center, Nijmegen, the Netherlands; 6 Department of Trauma Surgery, Amsterdam UMC, Vrije Universiteit, Amsterdam, the Netherlands; 7 Department of Trauma Surgery, Maastricht University Medical Centre, Maastricht, the Netherlands; 8 Department of Forensic Medicine, GGD Euregio, Enschede, the Netherlands; 9 Department of Surgery, Haga Teaching Hospital & Juliana Children’s Hospital, The Hague, the Netherlands; 10 Department of Forensic Medicine, GGD Utrecht, Zeist, the Netherlands; 11 GGD Zuid-Limburg, Department of Forensic Medicine, Maastricht, the Netherlands; 12 Department of Forensic Medicine, GGD Haaglanden, The Hague, the Netherlands; 13 GGD Hollands-Midden, Department of Forensic Medicine, Leiden, the Netherlands; 14 Department of Surgery, Division of Paediatric Surgery, University Medical Centre Groningen, Groningen, the Netherlands; 15 Erasmus Medical Centre, Department of Paediatric Surgery, Sophia Children’s Hospital, Rotterdam, the Netherlands; 16 Department of Trauma Surgery, Leiden University Medical Centre, Leiden, the Netherlands; 17 Forensisch Artsen Rotterdam-Rijnmond’ (FARR), Rotterdam, the Netherlands; 18 GGD Groningen, Department of Forensic Medicine, Groningen, the Netherlands; 19 Department of Forensic Medicine, GGD Amsterdam, University of Amsterdam, Amsterdam, the Netherlands; 20 Department of Paediatric Surgery, University Medical Centre Utrecht, Utrecht, the Netherlands; 21 Isala Clinics, Department of Trauma Surgery, Zwolle, the Netherlands; 22 Amsterdam UMC, Department of Social Paediatrics, Emma Children’s Hospital, University of Amsterdam, Amsterdam, the Netherlands; 23 Department of Trauma Surgery, Elizabeth TweeSteden Hospital, Tilburg, the Netherlands; 24 GGD Brabant, Department of Forensic Medicine, Tilburg, the Netherlands; 25 Department of Forensic Medicine, Section On Forensic Paediatrics, Netherlands Forensic Institute, The Hague, The Netherlands; 26 Department of Trauma Surgery, Medisch Spectrum Twente, Enschede, the Netherlands; 27 Amsterdam UMC, Department of Radiology and Nuclear Medicine, Emma Children’s Hospital, University of Amsterdam, Amsterdam, The Netherlands; 28 Amsterdam Center for Forensic Science and Medicine, Amsterdam, The Netherlands; 29 SpoedZorgNet AMC, Amsterdam UMC, Amsterdam, The Netherlands; 30 Forensic Medicine Department, GGD Amsterdam, Amsterdam, The Netherlands; 31 Department of Paediatrics, Amsterdam UMC, Amsterdam, The Netherlands; 32 Department of Trauma Surgery, Amsterdam UMC, Amsterdam, The Netherlands; 33 Onze Lieve Vrouwe Gasthuis, Amsterdam, The Netherlands; 34 Masterstudent Medicine, University of Amsterdam, Amsterdam, The Netherlands; 35 Bachelorstudent Forensic Science, Hogeschool van Amsterdam, Amsterdam UMC, Amsterdam, The Netherlands; 36 Department of Social Paediatrics, Amsterdam UMC, Amsterdam, The Netherlands; 37 Netwerk Acute Zorg Noordwest, Amsterdam, The Netherlands; 38 Department of Social Paediatrics, University Medical Center Rotterdam, Rotterdam, The Netherlands; 39 Traumacentre Southwest Netherlands, Rotterdam, The Netherlands; 40 Department of Paediatric Surgery, University Medical Center Rotterdam, Rotterdam, The Netherlands; 41 Department of Pediatric Orthopedics, University Medical Center Rotterdam, Rotterdam, The Netherlands; 42 Department of Trauma Surgery, University Medical Center Rotterdam, Rotterdam, The Netherlands; 43 Netwerk Acute Zorg Brabant, Tilburg, The Netherlands; 44 Department of Paediatric Surgery, Haga Ziekenhuis, Den Haag, The Netherlands; 45 Netwerk Acute Zorg regio West, Leiden, The Netherlands; 46 Netwerk Acute Zorg regio Zwolle, Zwolle, The Netherlands; 47 GGD Hollands-Midden, LUMC, Leiden, The Netherlands; 48 Department of Paediatrics, Leids Universitair Medisch Centrum, Leiden, The Netherlands; 49 Netwerk Acute Zorg Euregio, Amsterdam, The Netherlands; 50 Netwerk Acute Zorg Limburg, Maastricht, The Netherlands; 51 Department of Paediatrics, Maastricht Universitair Medisch Centrum, Maastricht, The Netherlands; 52 Department of Traumasurgery, Maastricht Universitair Medisch Centrum, Maastricht, The Netherlands; 53 Division of Special Services, NFI, The Hague, The Netherlands; 54 Division of Special Services, Section Forensic Pathology, NFI, The Hague, The Netherlands; 55 Department of Trauma Surgery, Radboudumc, Nijmegen, The Netherlands; 56 Department of Surgery, Division of Trauma Surgery, UMCG, Groningen, The Netherlands; 57 Emergency Care Network Northern Netherlands (AZNN), Groningen, The Netherlands; 58 Department of Trauma Surgery, University of Groningen, University Medical Center Groningen, Groningen, The Netherlands; 59 Traumazorgnetwerk Midden-Nederland, UMCU, Utrecht, The Netherlands; 60 Department of Social Paediatrics, UMCU, Utrecht, The Netherlands

**Keywords:** Non-accidental trauma, Child abuse, Neglect, Postmortem investigation, Deceased, Child

## Abstract

**Purpose:**

Between 0.1—3% of injured children who present at a hospital emergency department ultimately die as a result of their injuries. These events are typically reported as unnatural causes of death and may result from either accidental or non-accidental trauma (NAT). Examples of the latter include trauma that is inflicted directly or resulting from neglect. Although consultation with a forensic physician is mandatory for all deceased children, the prevalence of fatal inflicted trauma or neglect among children is currently unclear.

**Methods:**

This is a retrospective study that included children (0–18 years) who presented and died at one of the 11 Level I trauma centers in the Netherlands between January 1, 2014, and January 1, 2019. Outcomes were classified based on the conclusions of the Child Abuse and Neglect team or those of forensic pathologists and/or the court in cases referred for legally mandated autopsies. Cases in which conclusions were unavailable and there was no clear accidental cause of death were reviewed by an expert panel.

**Results:**

The study included 175 cases of childhood death. Seventeen (9.7%) of these children died due to inflicted trauma (9.7%), 18 (10.3%) due to neglect, and 140 (80%) due to accidents. Preschool children (< 5 years old) were significantly more likely to present with injuries due to inflicted trauma and neglect compared to older children (44% *versus* 6%, *p* < 0.001, odds ratio [OR] 5.80, 95% confidence interval [CI] 2.66–12.65). Drowning accounted for 14 of the 18 (78%) pediatric deaths due to neglect, representing 8% of the total cases. Postmortem radiological studies and autopsies were performed on 37 (21%) of all cases of childhood death.

**Conclusion:**

One of every five pediatric deaths in our nationwide Level I trauma center study was attributed to NAT; 44% of these deaths were the result of trauma experienced by preschool-aged children. A remarkable number of fatal drownings were due to neglect. Postmortem radiological studies and autopsies were performed in only one-fifth of all deceased children. The limited use of postmortem investigations may have resulted in missed cases of NAT, which will result in an overall underestimation of fatal NAT experienced by children.

**Supplementary information:**

The online version contains supplementary material available at 10.1007/s12024-021-00416-7.

## Introduction

In 2003, the United Nations Children’s Fund reported that at least 3,500 children residing in high-income countries die annually as a result of non-accidental injuries (NAI) [1]. The prevalence of child abuse in the Netherlands has been estimated to be between 90,000 – 127,000 cases annually [2, 3]. According to Statistics Netherlands, there are approximately 3.4 million children currently living in the Netherlands [4] and 1,000–1,100 child deaths each year [5].

**Fig. 1:**
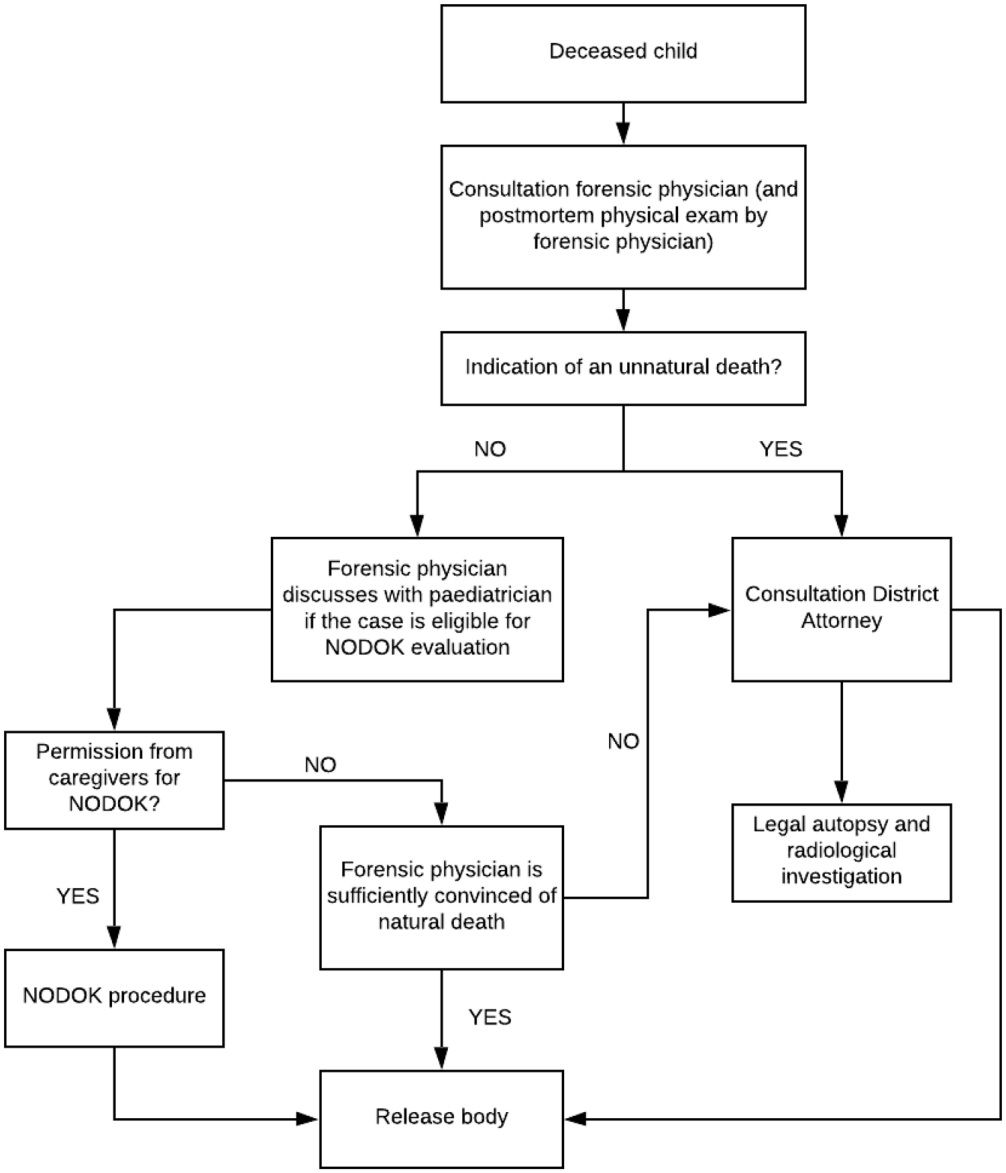
The Dutch medico-legal system of postmortem investigation in children. In the Netherlands, the DA decides whether postmortem investigations should be performed. This decision is based on information from the police and forensic physician. The forensic physician examines the child externally, collects the clinical history and results of investigations performed at the hospital. Postmortem legal investigations (autopsy, imaging, toxicology) are requested by the DA after consultation of the forensic physician and police and based on their information the cause of death is highly suspected for a crime. The forensic physician is a medical doctor specialized in forensic medicine (duration of forensic medicine specialization is 3 years). The DA has a bachelors and master’s degree in (criminal) law together with an additional training at the public prosecutors office of 4 years. Few DA’s are specialized in child abuse. Cases of pediatric deaths are handled by the DA on call (who may or may not be specialized in child abuse).

From 1996 to 2009, 445 cases of childhood death were referred to the Netherlands Forensic Institute (NFI) for mandatory autopsies; 239 of these children died due to NAI [6], corresponding to an average of 17 cases of fatal NAI per year. However, this is likely to be an underestimation resulting from selection bias. Some cases that were categorized as natural death might have been recognized as unnatural death due to NAI if a full postmortem investigation had been performed. Article 2 of the Council of Europe Committee of Ministers (CECM) statement on the harmonization of rules governing medicolegal autopsies states clearly that autopsies should be carried out in all cases of obvious or suspected unnatural death in both children and adults [7, 8]. While recommendations suggest that these principles should be implemented by all member states of the European Union, adoption of the entire set of guidelines is not currently mandatory. Moreover, member states are permitted to design their own systems based on these guidelines. Article 2 of the CECM has not been ratified in the Netherlands. The system currently in place in the Netherlands focuses on the results of a complete physical examination of the deceased; postmortem radiological imaging studies or legally mandated autopsies are only performed if there are suggestions or strong indications that death was a result of a criminal act. In contrast to cases of adult deaths, a forensic physician must be consulted in all pediatric deaths before a certificate of death can be issued. A consultation can result in one of three possible scenarios (Fig. 1). In the first scenario, there will be a clear indication of an unnatural cause of death, a situation that may lead to a full medicolegal evaluation. The second scenario addresses an expected, i.e., natural cause of death, for example, death of a child in palliative care due to the progression of a metastatic tumor. In this case, the body is released to the caregivers. The third scenario addresses cases in which the cause of death remains unclear. In these cases, a non-mandatory postmortem evaluation (as per the “Nader Onderzoek naar de DoodsOorzaak van Kinderen” [NODOK] or “Further Examination of the Cause of Death in Children” procedure) is offered to the caregivers [9–11].

Between 0.1–3% of injured children admitted to the Emergency Department (ED) ultimately die as a result of their injuries (i.e., experience an unnatural death) [12, 13]. After consultation with the attending physician, the forensic physician will discuss the events leading to the presentation at the ED and the findings of the postmortem physical examination with the district attorney (DA). The decision as to whether the body can be released to the caregivers or taken into custody for a legally mandated autopsy lies solely with the DA. All legally mandated autopsies will be performed at the Netherlands Forensic Institute (NFI). The decision is based primarily on whether a given death was the result of a criminal act. In other words, postmortem investigations in unnatural deaths identified in the Netherlands are primarily initiated to rule out a crime, rather than to explain the cause of death.

Despite required consultations with forensic physicians, the prevalence of non-accidental death remains unclear. Equally important, the characteristics of fatal NAI in children have not been fully identified.

This study aimed to answer the following research questions:

(1) What is the prevalence of non-accidental trauma (NAT) in children who were admitted to and died at Level I trauma centers (TCs) in the Netherlands?

(2) What are clinical and postmortem features of non-accidental and accidental death in children who were admitted to these Level I TCs?

## Methods

### Study design

This was a retrospective study of children who presented and died at one of the 11 Level I TCs in the Netherlands (see Acknowledgments) between January 1, 2014, and January 1 2019. This study was carried out by the nationwide collaborative group known as ASANTE (which is the Kiswahili word for thank you). All hospitals were designated as Level I TCs as defined by the American College of Surgeons Committee on Trauma [14]. Additional patient data were retrieved from databases of the Dutch public health services (GGD), the Forensisch Artsen Rotterdam-Rijnmond (a private forensic medical organization), and the Department of Forensic Medicine, Netherlands Forensic Institute (NFI).

The Medical Ethics Review Committee (MEC) of the Academic Medical Center, Amsterdam reviewed this study (reference number W18_319 #18.366 dated October 4, 2018) and concluded that no informed consent was required in accordance with national and international legislation. Boards and MECs of all participating hospitals also reviewed and approved the study protocol. Data processing and storage were in accordance with European Union General Data Protection Regulation. Data were collected using an electronic data capture system (Castor EDC, 2019, Amsterdam, the Netherlands) [15].

Eligibility criteria for this study are provided in Table 1.

**Table 1:** Eligibility criteria

*Inclusion*	
	Children aged 0 – 18 years
	Admission to a Level I trauma centre in the Netherlands
	Deceased either at admission or during subsequent hospitalization
	Unnatural cause of death
	Presentation at shockroom due to: trauma, drowning and resuscitation (sudden cardiopulmonary arrest)
*Exclusion*	
	Referral to hospitals outside the Netherlands immediately after primary survey and stabilisation of the patient Suicide
	Indeterminate cases

### Definitions and outcomes

The primary outcome of this study was the prevalence of non-accidental trauma (NAT) among children who presented and died at Level I TCs in the Netherlands.

Secondary outcomes include:

(1) Indicators of fatal NAT (inflicted or secondary to neglect) compared with fatal accidental trauma (AT) in children, including clinical history, injuries, and demographics.

(2) Causes of death determined by forensic evaluations.

(3) Performance of postmortem investigations.

The definition of NAT (i.e. trauma inflicted directly or caused by neglect) used in this study is based on the law in the Netherlands and includes any form of physical interaction that is threatening or violent to a minor, actively or passively urged by the caregivers or other persons towards whom the minor is in a relationship of dependence or lack of freedom, that results in serious harm or the threat of serious harm to the minor in the form of physical injury. This is consistent with the definition of maltreatment formulated by the World Health Organization [16, 17].

Non-accidental injuries (NAIs), which include injuries due to NAT [16, 17], including the following:

1. Inflicted trauma: injuries resulting from the force of impact via the direct actions of someone other than the child him or herself, regardless of motive.
2. Neglect: injuries resulting from the failure to protect a child from physical harm. Neglect was scored based on two domains, including supervision and environment. In other words, if either the environment or supervision were deemed to be inadequate, the case was classified as neglect (Appendix A includes a detailed description of these parameters).
3. Accidental trauma (AT): injuries in which there was no reasonable suspicion of inflicted trauma or neglect. The trauma mechanism was considered to be accidental by society at large and based on standard unwritten laws and definitions. In these cases, trauma was frequently observed by a person other than the primary caregivers (i.e. a “witnessed accident”) and did not raise any concern for NAT.
4. Indeterminate: injuries in which there was insufficient information available. In these cases, the cause of injuries sustained could not be determined and it was not possible to classify the case in one of the three aforementioned categories.

The Child Abuse and Neglect-team (CAN-team) is a multidisciplinary team specialized in the evaluation of suspected cases of child abuse. The team usually includes a pediatrician who has specialized in social pediatrics, a pediatric radiologist, a pediatric surgeon, an ED physician or nurse, and a physician from the national Child Protective Services (“Veilig Thuis”, or CPS). Some of the hospitals maintained permanent CAN-teams that included a pediatrician specialized in social pediatrics, an ED physician or nurse, and several social workers. In these cases, a trauma or pediatric surgeon, a radiologist, and CPS were consulted if necessary.

An external postmortem examination is a physical exam carried out on the body by the forensic physician [8]. Likewise, postmortem investigations can include radiologic and clinical findings and results from legally mandated autopsies, toxicology screening, and ophthalmologic investigations. A forensic evaluation is an assessment of all information collected including the external forensic examination as well as clinical and postmortem investigations (the former obtained before death) and police information.

### Classification of cases

Conclusions from the CAN-teams, forensic pathologists (in cases of legally mandated autopsies), the courts, and/or the expert panel were used to classify AT and NAT. Cases were classified into one of three groups; AT, NAT (subdivided into inflicted trauma or neglect), or indeterminate. All cases with a clear accidental cause (i.e. those observed by a third party and were not associated with concerns regarding inflicted trauma or neglect) were classified as AT. All cases evaluated by hospital-based CAN-teams were assessed further. Cases were referred to Child Protective Services (CPS) when a CAN-team concluded that a child was injured due to (suspected) NAT. Conclusions of the CAN-teams were used to classify patients into one of the three subcategories. Some cases were referred to CPS without input from the CAN-teams; feedback from CPS was used to classify these cases. The third group included cases in which the cause of injury was not evident and no CAN-team was available. These cases were evaluated by an expert panel that consisting of five members of the research group, including a trauma surgeon (EK), a pediatric surgeon (RB), a forensic pediatric radiologist (RvR), a forensic physician (SdV), and a pediatrician who specialized in child abuse (AHT). The expert panel discussed each of these cases and determined whether the injuries were accidental *versus* potentially inflicted or caused by neglect. Because the expert panel was reviewing these cases retrospectively, it was not always possible to draw definite conclusions regarding inflicted trauma based on the available information. Some cases lacked relevant additional information. These cases were classified as indeterminate.

### Data extraction

We collected detailed clinical data, psychosocial information, findings from the CAN-teams, and the results of the postmortem physical examinations using a standardized case report form maintained in an electronic database (Castor EDC, 2019, Amsterdam, the Netherlands) [15]. Identification of eligible patients, information regarding demographics, and length of stay at the hospital were provided by the Dutch national trauma registration [18]. Clinical information included a description of all identified injuries, a detailed history and injury mechanism, information as to whether surgical intervention was required and whether surgery was performed together with details of the child abuse screening at the ED [19]. Postmortem information included a description of all injuries identified in postmortem examinations, causes of death, radiological investigations, and the outcomes of legally mandated autopsies or “Nader Onderzoek naar de DoodsOorzaak van Kinderen” (NODOK), which translates to “Further Examination of the Cause of Death in Children”, which is a stepwise approach used to investigate the cause of death in children who may have experienced an unexpected or unexplained death [1, 11]. Social information included details concerning socio-economic status based on postal code data provided by Statistics Netherlands. Socioeconomic status was listed in tertiles ranging from (1) low to (3) high [20] together with family composition. Information provided by the CAN-teams included conclusions as to whether NAT was suspected and if further actions were required (e.g., consultation with social services, care under supervision, foster care, reporting to police, among others). The NFI provided information on the cause of death in cases referred for legally mandated autopsy.

### Statistical analysis

Data were analyzed using SPSS version 27 (IBM Corp., Armonk, NY, USA). Quantitative variables were summarized as medians and interquartile ranges, while categorical variables were presented as counts and percentages. Three groups were used for the analysis: inflicted trauma, trauma due to neglect, and accidental trauma. Subgroup analyses were performed for young children (< 2 years old), preschool children (< 5 years old) and older children (≥ 5 years old). The Chi-square test/Fisher’s exact test and Kruskal–Wallis test were used to identify statistically significant differences. Odds ratios (ORs) were calculated using crosstabs. A *p*-value less than 0.05 was considered statistically significant; 95% confidence intervals (CIs) were calculated as appropriate.

## Results

### Case classification

**Fig. 2:**
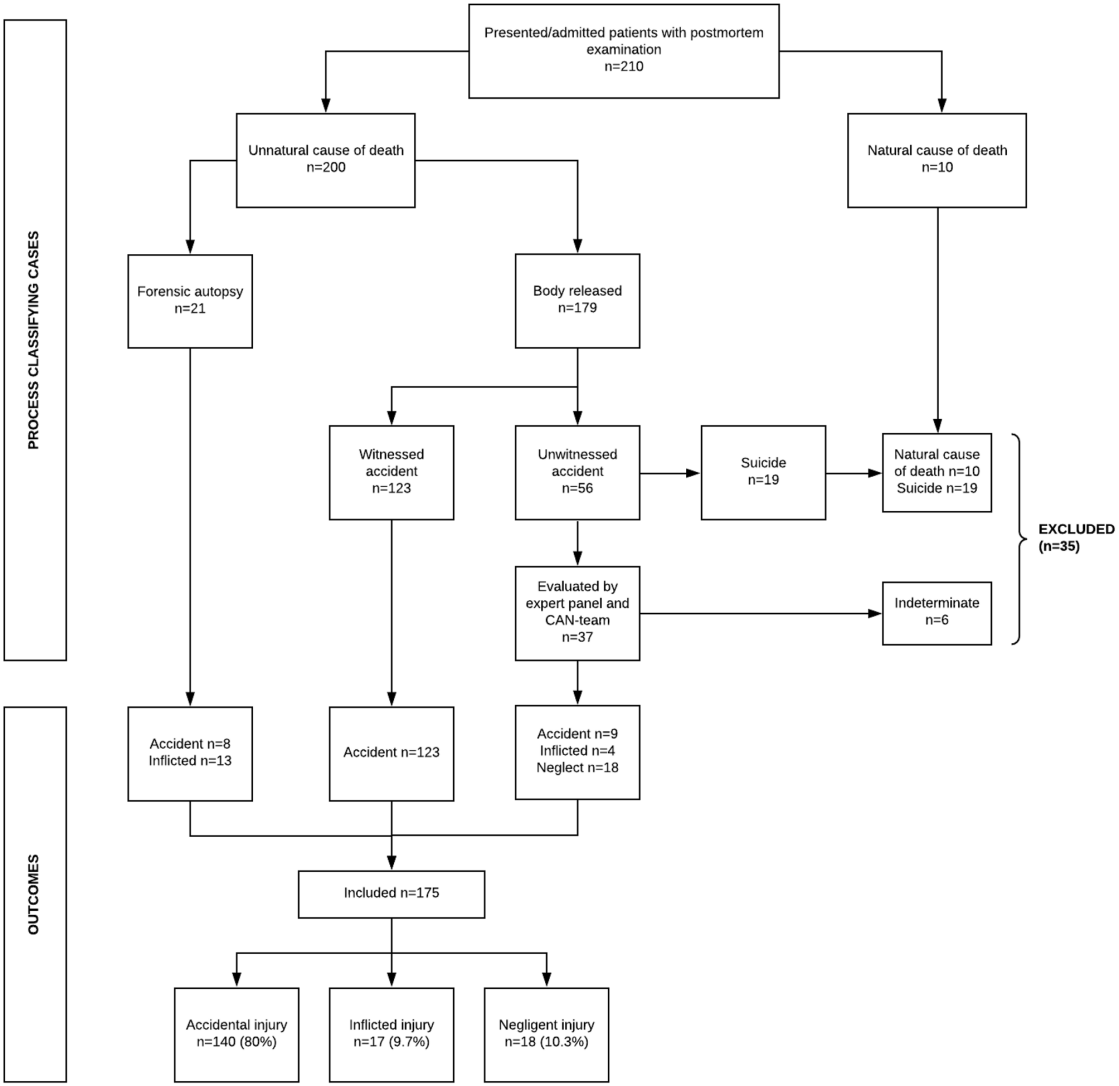
Classification of cases of the total study group.

Two hundred and ten children were admitted to a Level I TC between January 2014 and January 2019 and died in the ED or after admission. All 210 cases underwent an external postmortem examination by a forensic physician. Twenty-one cases (10%) were referred for legally mandated autopsy. The remaining cases (n = 179, 90%) were released to their respective caregivers (Fig. 2). Fifty-one of these cases (24%) underwent postmortem investigations (radiology, clinical autopsy, toxicology). As a result, 10 children (5%) were found to have died of natural causes; suicide was confirmed in an additional 4 cases (2%). Natural deaths, suicides, and indeterminate cases were excluded from the analysis. After exclusion, a total of 175 cases were included in our analysis. An overview of these 175 cases is presented in Fig. 2.

Case 1 A thirteen-month old girl was admitted to the ED due to a sudden cardiopulmonary arrest. That morning, she had been taken to the nanny and, except for a running nose and a sub-febrile temperature, seemed to be fine. Half an hour after feeding the girl, the nanny found her unresponsive in her crib and she contacted the emergency services immediately. The girl was unsuccessfully resuscitated at the hospital. After she passed away, the forensic physician was consulted and did not witness any injuries or bruises during the external postmortem examination. Although the hypothesis was that the child died of a possible viral infection, the DA requested a postmortem CT-scan to rule out other causes of death*. Before the CT-scan was performed, the organs were removed for donation (with permission of the DA) and no abnormalities of the organs were detected. The CT-scan showed a healing fracture of the right humerus, upon which the girl’s body was seized for further evaluation and brought to the NFI for a legal autopsy. The autopsy revealed additional fractures and extensive thin film subdural hematoma (which was not visible on the CT-scan).

* This is exceptional because usually no postmortem investigations are conducted in such a case without any signs of unnatural death.

### Children dying of unnatural causes

An overview of demographic data and trauma mechanisms is provided in Table 2. The 175 cases of children who died of unnatural causes included 114 (65%) boys with a median age of 7.5 years (interquartile range [IQR] 2–15 years) and 61 (35%) girls with a median age of 9 years (IQR 2.5–14 years). Seventeen (9.7%) children died of inflicted injuries and 18 (10.3%) of neglect. In 140 (80%) cases, the cause of death was identified as accidental (Fig. 2). Children with inflicted (median age, 5 months; IQR 2–31 months) and negligent injuries (median age 42 months, IQR 30–63 months) were significantly younger than those succumbing to accidental injuries (median age 136 months, IQR 58–189 months, *p* < 0.001; Table 2). Children succumbing to inflicted injuries were more frequently those living in either lower (*p* = 0.019) or higher socioeconomic neighborhoods (*p* = 0.022; Table 2).

**Table 2:** Demographic information and trauma mechanism

	**Inflicted** **n = 17 (9.7%)**	Neglect n = 18 (10.3%)	Accidental n = 140 (80%)	*p*-value	Total n = 175 (100%)
**Age, months (median, IQR)**	5 (2 – 31)	42 (30 – 63)	136 (58 – 189)	< 0.001*	101 (32 – 178)
**Boys (n,%)**	9 (53)	14 (78)	91 (65)	0.306	114 (65)
Age, months (median, IQR)	2 (1.5 – 5)	43 (30 – 58)	146 (43 – 194)	< 0.001*	94 (31 – 185)
**Girls (n,%)**	8 (47)	4 (22)	49 (35)	0.306	61 (35)
Age, months (median, IQR)	13 (8 – 77)	53 (20 – 103)	128 (64 – 179)	0.010*	116 (37 – 174)
**SES (n,%)**					
Low	7 (41)	4 (22)	20 (14)	0.019*	31 (18)
Average	4 (24)	14 (78)	97 (69)	< 0.001*	115 (66)
High	5 (29)	-	16 (11)	0.022*	21 (12)
Unknown	1 (6)	-	7 (5)	-	8 (5)
**Cause (n,%)**					
Traffic accident	-	2 (11)	78 (56)	< 0.001*	80 (46)
Drowning	-	14 (78)	18 (13)	< 0.001*	32 (18)
Suffocation	2 (12)	-	16 (11)	0.318	18 (10)
Blunt trauma	-	-	11 (8)	0.233	11 (6)
Fall from height	2 (12)	1 (6)	8 (6)	0.620	11 (6)
Sudden cardiopulmonary arrest	11 (64)	1 (6)	5 (4)	< 0.001*	17 (10)
Sharp penetrating trauma	2 (12)	-	2 (1)	0.022*	4 (2)
Explosion	-	-	1 (< 1)	-	
Unknown	-	-	1 (< 1)	-	
**Length of stay (median, IQR)	2 (1.5 – 4)	1 (1 – 3)	2 (1 – 3)	0.162	2 (1 – 3)
**ICU-admission (n,%)** **Length of stay ICU (median, IQR)	15 (88) 2 (2 – 4)	7 (39) 3 (2 – 11)	105 (75) 2 (2 – 5)	0.002* 0.503	127 (73) 2 (2 – 5)

^*^ Statistical significance, SES = socio-economic status, **Length of stay in days, ICU = Intensive Care Unit

### Trauma mechanisms and injuries

The most prevalent mechanisms associated with lethal trauma were traffic accidents (*n* = 80, 46%) and drownings (*n* = 32, 18%). Traffic accidents accounted for most childhood deaths in the AT group (56%), compared to 0% in the group that included inflicted trauma and 11% among those categorized as neglect (*p* < 0.001). Drowning was the main cause of death (78%) in cases of NAT due to neglect, compared to 0% of the inflicted NAT cases and 13% in the AT group (*p* < 0.001). Seventeen children were presented with “sudden cardiopulmonary arrest” without apparent injuries. Injuries resulting in cardiopulmonary arrest in these cases were identified during resuscitation or postmortem evaluation. Admission due to sudden cardiopulmonary arrest without apparent injuries was identified at a significantly greater frequency (*p* < 0.001) among those in the NAT inflicted group (*n* = 11, 64%) compared with the cases of NAT due to neglect (*n* = 1, 6%) and those in the AT group (*n* = 5, 4%).

Table 3 provides an overview of different types of injuries within the three groups with calculated ORs. Traumatic brain injury was the most prevalent type of injury in the NAT inflicted group (88%) and the AT group (76%); this diagnosis was identified significantly less frequently among those in the NAT neglect group (39%, *p* = 0.001). Fractures, including rib fractures, were frequently identified in both the NAT inflicted group and AT group (47% and 61%, respectively), but were detected significantly less frequently among those with NAT due to neglect (11%; *p* < 0.001). No differences in types of injury were identified in comparisons between cases of inflicted NAT and AT. Intrathoracic injuries were not detected in any of the NAT inflicted cases compared with 36% among those in the AT group; OR 0.84). By contrast, bruises had a significantly higher OR among cases with inflicted NAT (41%) versus AT (8%; OR 8.21).

**Table 3:** Injuries of all deceased children

	**Inflicted** **n = 17**	**Neglect** **n = 18**	**Accidental** **n = 140**	**p-value**	**OR (95%CI)** **Abuse vs Accident**	**OR (95%CI)** **Neglect vs Accident**
**Injuries**						
Traumatic brain injury	15 (88)	7 (39)	106 (76)	0.001*	2.41 (0.52–11.06)	0.20 (0.07–0.57)*
Fracture	8 (47)	2 (11)	85 (61)	< 0.001*	0.58 (0.21–1.58)	0.08 (0.02–0.37)*
Intra thoracic injury	-	4 (22)	51 (36)	0.007*	0.84 (0.77–0.91)*	0.50 (0.16–1.60)
Intra-abdominal injury	2 (12)	2 (11)	31 (22)	0.368	0.47 (0.10–2.16)	0.44 (0.10–2.01)
Aspiration/ARDS	-	3 (17)	13 (9)	0.232	-	1.95 (0.50–7.65)
Bruises	7 (41)	-	11 (8)	< 0.001*	8.21 (2.61–25.81)*	**-**
Laceration	2 (12)	-	17 (12)	0.296	0.97 (0.20–4.59)	**-**
Other**	-	-	4 (3)	-	-	-

^**^Remaining injuries: spinal injury (2x), dissection of carotid artery (1x), retinal haemorrhage (1x)

### Postmortem investigations

Postmortem radiologic studies without autopsy were conducted in 12 (7%) cases, while combined legally mandated autopsy and radiologic studies were performed in 19 (11%) cases (Table 4). Postmortem investigations were performed on a total of 37 (21%) of the cases included in the study sample. This included investigations performed in 15 (88%) of the cases of non-accidental inflicted trauma, in 3 (17%) of the cases of NAT associated with neglect, and 19 (14%) of those in the AT group. Occult injuries were detected in 6 children in the NAT inflicted group, compared to only one case of NAT due to neglect and 3 among these in the AT group. Of note, the occult injuries identified in the single NAT due to neglect case raised no concern regarding the potential for inflicted injury.

**Table 4:** Postmortem investigation

		**Inflicted** **n = 17**	**Neglect** **n = 18**	**Accidental** **n = 140**
**Postmortem investigation (N,%)**		15 (88)	3 (17)	19 (14)
**Type of postmortem investigation**				
	Radiology*	2	3	7
	Legal autopsy	-	-	1
	Clinical autopsy	-	-	5
	Radiology & legal autopsy & toxicology	13	-	7
	Toxicology**	1	1	6
	Others***	2	-	1
**Occult injuries detected (n,%)**		6	1	3

^*^ Consisting of skeletal survey and total body CT-scan

^**^ Toxicology screening performed separately from the legal autopsy. Toxicology is performed as standard during a legal autopsy

^***^Other investigations: blood culture (2x) and ophthalmoscopy (1x)

### Subgroup analysis of preschool-aged children

Preschool-aged children (< 5 years old) were significantly more likely injured due to inflicted trauma and neglect compared to older children (44% *versus* 6%; *p* < 0.001, OR 5.80, 95% CI 2.66–12.65). Fatal NAT was identified as the cause of death for 28 (44%) of the preschool-aged children, of whom 15 (23%) succumbed to non-accidental inflicted trauma and 13 (20%) to NAT secondary to neglect. Fatal trauma mechanisms in preschool-aged children included sudden cardiopulmonary arrest (*n* = 16, 25%), drowning (*n* = 16, 25%), and suffocation (*n* = 13, 20%). In children younger than two years of age, NAI was identified in 15 (43%) cases as the cause of death. Fatal trauma mechanisms resulting in NAI were sudden cardiopulmonary arrest (*n* = 15, 43%), suffocation (*n* = 11, 31%), and drowning (*n* = 5, 14%). An overview of the classification of cases in each age group is presented in Table 5.

**Table 5:** Case classification stratified per age category

	**0 – 2 years old**	**2 – 5 years old**	** ≥ 5 years old**
Total (n,%)	35 (20)	29 (17)	111 (63)
Inflicted (n,%)	13 (37)	2 (7)	2 (2)
Neglect (n,%)	2 (6)	11 (38)	5 (4)
Accident (n,%)	20 (57)	16 (55)	104 (94)

## Discussion

We report here the 20% prevalence of fatal NAT among children admitted to a Level I TC. We showed that 9.7% of the cases succumbed to inflicted trauma and 10.3% to the consequences of neglect. Fatal NAT was identified significantly more frequently as the cause of death in preschool-aged children (< 5 years old) compared to children in the older age cohorts. Subgroup analysis in young children (< 2 years old) revealed a 37% prevalence of inflicted trauma and a 6% prevalence of neglect.

One important finding relates to the cause of inflicted deaths. Of the cases of inflicted trauma, 64% presented to the ED as “sudden cardiopulmonary arrest”. This finding has extremely important implications, as there may be cases of non-accidental cases of inflicted trauma presenting as “sudden death” that have not been evaluated appropriately. Consequently, cases of non-accidental inflicted trauma may have been ignored because not all cases of sudden death undergo postmortem investigations. Although the NODOK procedure is offered to caregivers, none of these investigations are mandatory. Autopsies are rejected by caregivers in three of ten cases. These findings might be considered in light of another recent study from the Netherlands in which 85% of NODOK cases underwent an autopsy [11]. The limited frequency of autopsies may lead to a misdiagnosis of sudden infant death syndrome (SIDS), or sudden unexpected death in infancy (SUDI) [21, 22]. A small fraction of reported SIDS/SUDI cases may be covert NAI; of note, the distribution of SIDS has remained stable over the years while preventive measurements have been drastically improved [23, 24]. For example, Cote et al. [25] reported that 2.6% of 623 deaths attributed to SIDS were actually due to NAI. They also note that the proportion of non-SIDS deaths was significantly higher (18% *versus* 6%) in centers with expertise in pediatric pathology. This highlights the importance of the standardization of postmortem evaluations used to diagnose pediatric deaths. This might be undertaken by professionals with expertise in pediatrics and NAI. The importance of standardization is illustrated by the case report included in Case 1. This case demonstrated clearly that a postmortem physical exam was not sufficient to diagnose abusive head trauma, which was found only after the child underwent a postmortem computed tomography scan, which led to a legally mandated autopsy.

Another reason to standardize postmortem evaluations is that the risk of missing NAI in young children is higher compared to older children, mainly because of physiological differences between these age groups. While babies and toddlers are developing muscle strength, their bony structures remain more malleable [26]. Subtle signs of trauma can be masked and may be externally invisible. Furthermore, blunt abdominal trauma can lead to severe intra-abdominal injuries without any bruises on the abdominal wall [27, 28] and intrathoracic injuries frequently remain undetected in postmortem physical examinations [29]. Twelve of the 21 children in our study had no visible external injuries at admission; all of these were children who were younger than two years of age. Injuries were identified in nine of these children via additional investigations in the ED (including traumatic brain injury, retinal hemorrhage, and healing rib fractures). Injuries leading to death were revealed by postmortem radiologic studies and autopsy in the remaining three children although they exhibited no external injuries (fractures, traumatic brain injury, or signs of suffocation). While fatal NAI can and does occur in older children, more force is needed to produce the same type of fatal injury compared to that needed to injure younger children.

Another important finding in our study was the proportion of children suffering from neglect. The main cause of negligent deaths was drowning (78%). Examples of these cases include infants left alone in the bathtub or young children permitted to play near a pond or swimming pool with interrupted supervision. Our aim is not to accuse caregivers; however, we identified a large group (10%) of children whose deaths could have been prevented if sufficient supervision and appropriate preventive measures were taken. The aim of identifying situations associated with negligence will be to engage in conversations with the broader society, optimize education, and increase awareness of these dangers with the goal of prevention. We observed that many caregivers overestimated the capabilities of children and/or underestimated the danger involved in a given situation. As physicians, we must acknowledge these dangers and provide counseling to caregivers. As shown by our data, the danger of water, notably in water-rich countries such as the Netherlands, was largely underestimated. Many of the drownings might have been prevented by placing a fence around the pool or pond. This is mandatory in some European countries. We highly recommend reevaluation of the current guidelines by the Dutch legislature to raise national awareness of this topic. This would also focus on improving the educational programs available for caregivers and immigrants.

## Strengths

This nationwide study comprises a large cohort of cases of unnatural pediatric deaths due to NAI and AI. We were able to generate a multidisciplinary overview of this population and fatal NAI in the Netherlands in general. The Dutch national trauma registration data collection is of high quality as is postmortem information collected by forensic physicians. Because of this collaboration, the data collected represented an amalgamation of clinical information from 11 hospitals and information from forensic physicians combined with findings from legally mandated autopsies provided by NFI. By merging these sources of information, we identified significant indicators of fatal NAI. In addition, our expert panel consisted of a balanced group of physicians from different specialties (including child abuse, pediatric surgery, trauma surgery, pediatric forensic radiology, forensic medicine, and pediatrics). We believe that all cases were assessed carefully by the members of this panel.

## Limitations

One limitation of this study is that we only collected data from cases of children who died while in a hospital, i.e. selection bias. To address this issue, we compared our results to the national death registration maintained by Statistics Netherlands [5]. A rough estimate revealed that approximately half of the children who succumbed to trauma did so outside a Level I TC. We hypothesize that these children most likely died due to major trauma associated with excessive force, for example, severe accidents (high-speed motor vehicle crashes), prolonged drownings, or inflicted trauma of a very serious nature (such as a fall from a great height). Moreover, we had to classify some cases as indeterminate because we did not have data that would permit the expert panel to reach specific conclusions. Furthermore, we were unable to identify household characteristics that might be associated with NAI. We were able to provide a rough estimate of socioeconomic status based on the patients’ postal code and residential neighborhood.

Another common limitation in the child abuse field is the lack of a diagnostic “gold standard”. We attempted to use a diagnostic-type method using the information provided by CAN-teams, forensic physicians, courtrooms, and the consensus opinions of the expert panel. While we considered the conclusions of the CAN-team to be accurate, in most cases we did not have any insight into the specific information used by the CAN-team to generate their conclusions. This limitation may have led to an overestimation of the population subjected to NAT. Moreover, both CAN-teams and the expert panel considered injuries in their assessment. While this may have resulted in circular reasoning, in some cases, investigations were limited and injuries could have been missed. Nonetheless, consensus opinions of expert panels have been identified as a reliable means of assessing cases [30].

Finally, we assume that we may have underestimated the prevalence of fatal NAI in this study because we have reasons to doubt the effectiveness of the postmortem system in the Netherlands for detecting NAI. It is critical to recognize that no postmortem investigations are conducted in cases in which there are no suspicions of criminal behavior (Fig. 1). However, postmortem analyses from these cases could provide valuable information regarding what may be undetected criminal acts. According to the European Autopsy rules of the Council of Europe Committee of Ministers (CECM) and the European Council of Legal Medicine (ECLM), while each member state needs to have an effective means to investigate deaths, each is provided with some independence regarding the design of this system [7]. Of course, every design has benefits and consequences. In the system currently in use in the Netherlands, the treating physician, the DA, and the forensic physician all play crucial roles in the determination of the cause and manner of death. Cases of NAI are likely missed because of a lack of awareness. Thus, the consequences of this system include the fact that we are at risk of missing non-accidental fatalities among our most vulnerable residents and thereby create insufficient awareness of this critical health problem. A recent NFI report documented that, based on decreasing numbers of autopsies, an estimated 20–25 murder cases were missed in the Netherlands in the year 2015 alone [31]. As noted in our study, the limited use of postmortem investigations may very likely have resulted in an underestimation of non-accidental cases of pediatric death.

Current law in the Netherlands requires a review of every pediatric death by a forensic physician. However, in practice, this is only done in ~ 70% of relevant cases [32]. This is another potential source of missed NAI cases, as it is clear that some cases were not reported to the forensic physician. Most cases underwent an external postmortem physical examination but no postmortem investigations (NB only 21% underwent postmortem investigations). As previously discussed, we advocate the more extensive use of postmortem investigations in cases of pediatric death. This exercise will be pivotal for diagnostic accuracy and for determining the cause and manner of death.

## Conclusion

In this nationwide Level I TC study, we identified NAI in 20% of children who experienced an unnatural death during a five-year period (January 2014 – December 2018). NAI was identified as the cause of 44% of the deaths in preschool-aged children (< 5 years old). Postmortem investigations were performed in only one of five children who died at the hospital. Of note, no data are available for the 50% of trauma-associated childhood deaths that took place outside a Level I TC. This may have resulted in an underestimate of the frequency of fatal NAI. Furthermore, our studies suggest strongly that a postmortem physical exam alone will not provide enough evidence to rule out the possibility of inflicted trauma in young children who have died as a result of unwitnessed trauma or under circumstances that are not fully clear. In these cases, we recommend more extensive postmortem investigations to rule out any possibility of inflicted death. Among these, cases of serial drownings should raise concerns regarding dangers in the environment and the potential lack of appropriate supervision. Educational programs focused on pediatric health and safety might be offered to caregivers by midwives, pediatricians, or general physicians.

## Key points

1. Non-accidental injuries were the cause of death of 20% of the patients in our pediatric cohort.
2. Non-accidental injuries were recognized as the cause of death in 44% of the children who were < 5 years of age.
3. Approximately 50% of non-accidental deaths in young children were due to inflicted trauma while 50% were due to neglect.
4. Most fatal drownings were the result of neglect and thus might have been prevented.
5. The limited use of postmortem investigations may have resulted in an underestimation of the prevalence of non-accidental death.

## Supplementary information

Below is the link to the electronic supplementary material.

Supplementary file1 (PDF 76 KB)
